# Evaluation of Unrestrained Replica-Exchange Simulations Using Dynamic Walkers in Temperature Space for Protein Structure Refinement

**DOI:** 10.1371/journal.pone.0096638

**Published:** 2014-05-21

**Authors:** Mark A. Olson, Michael S. Lee

**Affiliations:** 1 Department of Cell Biology and Biochemistry, Molecular and Translational Sciences, USAMRIID, Fredrick, Maryland, United States of America; 2 Advanced Academic Programs, Zanvyl Krieger School of Arts and Sciences, Johns Hopkins University, Baltimore, Maryland, United States of America; 3 Computational Sciences Division, U.S. Army Research Laboratory, Aberdeen Proving Ground, Maryland, United States of America; University of Michigan, United States of America

## Abstract

A central problem of computational structural biology is the refinement of modeled protein structures taken from either comparative modeling or knowledge-based methods. Simulations are commonly used to achieve higher resolution of the structures at the all-atom level, yet methodologies that consistently yield accurate results remain elusive. In this work, we provide an assessment of an adaptive temperature-based replica exchange simulation method where the temperature clients dynamically walk in temperature space to enrich their population and exchanges near steep energetic barriers. This approach is compared to earlier work of applying the conventional method of static temperature clients to refine a dataset of conformational decoys. Our results show that, while an adaptive method has many theoretical advantages over a static distribution of client temperatures, only limited improvement was gained from this strategy in excursions of the downhill refinement regime leading to an increase in the fraction of native contacts. To illustrate the sampling differences between the two simulation methods, energy landscapes are presented along with their temperature client profiles.

## Introduction

The structure-based discovery of small-molecule protein inhibitors and protein-derived biopharmaceuticals requires the structural target to be of sufficient resolution to capture accurate modeling details. For many druggable targets, the protein structure is unknown and, depending on sequence similarity with known crystallographic or NMR structures, can be anticipated by comparative modeling methods. Where structural templates are lacking for accurate sequence recognition and alignment, knowledge-based simulation methods can provide good to moderate resolution for particular classes of protein fold topologies [1], [2]. In either case, the protein models are decoys of the true native state and thus require some level of structural refinement to extend their all-atom resolution [3]–[14].

A common approach for the conformational sampling of decoy structures is the application of molecular dynamics parallel tempering, or commonly known as temperature-based replica exchange (T-ReX) [15]. Unlike traditional molecular dynamics simulations, T-ReX is a generalized ensemble method of applying multiple parallel simulations in which each replica is executed at a different temperature. In typical applications, the temperatures are pre-determined by a fixed set of values that span a desired range. While a fixed temperature distribution is thought to be ideal for many applications [16], it becomes pathological for cases where a sharp energy barrier separates conformational states [17]. The incurred difficulty arises from insufficient exchanges among nearest-neighbor replica clients at the so-called “phase transition” temperature. A sharp transition is common to modeling protein folding-unfolding events [17], although in general a highly frustrated energy landscape can hinder temperature swapping among clients.

Recently, we implemented an adaptive T-ReX algorithm based on the notion of enriching the population of clients and their exchanges near a protein folding-unfolding transition temperature by allowing the clients to dynamically walk in temperature space [18]. The implemented algorithm was first developed by Hansmann and coworkers [19], and Troyer and coworkers [20]. Our initial application of their method was modeling the folding-unfolding of SH3, a 57-residue protein domain of alpha-spectrin [18]. It was observed from our work that the adaptive T-ReX simulation method yielded a significantly lower melting transition temperature than the conventional static T-ReX approach, leading to a better agreement with the experimental determination. Although the adaptive method did not achieved proper thermodynamic coexistence between the folded and unfolded states, the improvement is thought to be gained from more extensive sampling of the transition state ensemble by allowing the replicas to circulate in temperature space, whereby visiting both low and high temperature extremes. An alternative adaptive algorithm has been developed based on convective methods to improve efficient sampling of energy basins that are limited by conventional replica-exchange methods [21].

While there are theoretical virtues and limitations of the adaptive T-ReX method, we investigate in this work the practical performance of the method applied to the structure refinement of a dataset of protein conformational decoys [7], [22]. Given the success of the adaptive T-ReX simulations for modeling the sharp energy differences between the folded and unfolded states of SH3 [18], it is of general interest to determine whether this approach is beneficial in modeling less-cooperative transitions that are thought to govern the structure refinement of decoys taken from either comparative modeling or knowledge-based structure predictions. We examine a set of protein targets that offers a challenging benchmark of simulation methods for structure refinement [7], [22]. The targets are 49–92 residues in size and amenable to an analysis by unrestrained all-atom simulation methods. Our simulation protocol is identical to that applied in earlier studies of refinement [7]. Computational sampling is conducted by combining the self-guided Langevin dynamics (SGLD) simulation method with replica-exchange simulations [23], [24], and we provide a comparison between the application of dynamic client walkers in temperature space and that of using a static distribution of temperatures. We also investigate four different energy functions to rank order conformations from the simulations.

## Methods

In conventional T-ReX simulations, the temperatures of the replica clients are fixed at specific values during the simulations and are typically geometrically spaced [16] by *N-*1 intervals from the minimum temperature *T*_min_ to the maximum *T*_max_

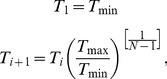
(1)where *i* = 1…*N* and the number of replica clients is given by *N*. After a specified number of simulation integration time steps, the neighboring replica clients, *a* and *b*, swap temperatures with a probability given by the Metropolis energy criteria [25]

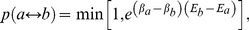
(2)where *β_a_* = 1/*k_B_T_a_*, *k_B_* is Boltzmann’s constant, *T_a_* is the temperature of replica client *a*, and *E_a_* is the potential energy of client *a*.

In contrast to Eq. (1), the adaptive T-ReX attempts to maximize the number of times that replica clients progress in round trips from the temperature extremes of *T*_min_ to *T*_max_. We define each replica client as either “cold” or “hot” depending on the last temperature extreme it visited [18]. Histograms over temperature space, 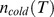 and 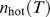, accumulate the number of cold and hot clients, respectively, visiting each temperature window. The fraction cold, *f*, of a client window at temperature *T* is the number of cold clients visiting that temperature divided by the total number of cold and hot client visits:
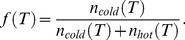
(3)


Using this fraction, we define a thermal current by [18]

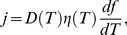
(4)where 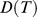 is the diffusivity and 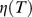 is the probability that any client will reside at temperature *T*. The current can be maximized by adjusting the temperatures such that *f* (*T_i_*) increases linearly as a function of temperature index, *i* (namely, the slope of *f* (*T_i_*) as a function of client index is constant) [18]. Here, we interpolate a continuous 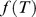 from the computed values of *f* at the current set of temperatures, *T_i_*, and then search for the new temperatures where 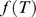 = *i*/(*N*−1). The effect of this procedure is that many temperatures will reside near a sharp energy barrier between conformational states. To prevent all of the windows from clustering around the same temperature and depleting exchanges at the temperature extremes, we introduce a constraint such that no neighboring temperatures can be more than two geometric spacing units apart,
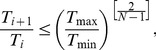
(5)with the lower and upper values of *T_i_* set to *T*_min_ and *T*_max_, respectively.

The starting input structures for the protein targets were obtained from the I-TASSER decoy set II of structurally non-redundant targets [26]. Selected targets for refinement were 1kviA, 1pgx, 1cy5A, 1shfA, 1r69, 1csp, 1ah9 and 1b72A. For each target, the I-TASSER decoys were ranked based on their calculated energies using the random-walk statistical potential (RWplus) developed by Zhang and Zhang [27]. The top-ranked 16 decoys per target were selected and their side chains were replaced using the SCWRL4 modeling program [28]. All selected decoys plus the corresponding X-ray crystal structures were subjected to energy minimization by the method of steepest descent minimization for 50 steps using the CHARMM22 force field with the CMAP backbone dihedral cross-term extension potential [29]. Solvent effects were modeled using the generalized Born (GBMV2) implicit solvent model [30]. The GBMV2 parameters were set to values of β = –12 and P3 = 0.65 to smooth the energy surface. The hydrophobic cavitation term was modeled by applying the solvent-exposed surface area of the protein solute with a surface tension coefficient set to a value of 0.015 kcal/mol/Å^2^.

The SGLD simulations were carried out using the CHARMM22+CMAP/GBMV2 potential energy function. Within the SGLD method, an *ad hoc* force term is computed as momentum averaged over the adjoining protein conformational space near the current conformation of the simulation trajectory. In the formalism of Wu and Brooks [23], the SGLD equation of motion is the following
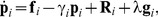
(6)where 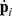 is the rate of change of the momentum of particle *i*, 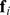 is the force acting on the particle, 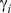 is the friction constant, 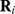 denotes the random force and 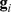 is a memory function, which is scaled by guiding factor λ. The memory function 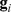 is defined by the moving average of the momentum seen by the system over an interval of time, *L*:
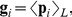
(7)where 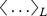 denotes a local average. The time interval is further defined as 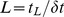, where 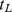 is the local averaging time and 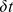 the time step along the simulation trajectory.

An integration time step of 2 fs was used for all simulations. SGLD parameters of the friction constant was set to γ of 1 ps^−1^ for all heavy atoms, the guiding factor λ set to a value of 1, and the averaging time 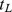 was set to 1 ps. Selection of these values was taken from our previous studies of the SGLD model [7], [22]. Non-bonded interaction cutoff parameters for electrostatics and vdW terms were set at a radius of 22 Å with a 2-Å potential switching function. Covalent bonds between the heavy atoms and hydrogen atoms were constrained by the SHAKE algorithm [31]. All protein targets during the simulations were unconstrained, freely to reorganize from conformational sampling.

Replica-exchange simulations were performed using the MMTSB [32] utilities and programming libraries for implementing the CHARMM simulation program (version c33b2) [33]. Simulations were carried out using 16 replica clients and frequency of exchanges was set to every 1 ps of simulation. As with previous structure refinement studies [7], the lower and upper bound temperatures were set at *T*_min_ = 275 K and *T*_max_ = 350 K. Selection of *T*_max_ was approximate of folding-unfolding transition temperatures and limits the unproductive use of multiple replica clients in oversampling the ensemble of unfolded states which contribute little to refinement. The static distribution of temperatures was spaced geometrically between the lower and upper limits. Each target was modeled for 20 ns of simulation time, which was found to be adequate to allow for sampling convergence of the decoys [7]. Conformational sampling results using the static T-ReX method for targets1kviA, 1pgx, 1cy5A, 1shfA, 1r69, and 1b72A were taken from earlier work [7].

Four different energy functions were applied to select the top-scoring conformers from the ensemble of decoys generated by the SGLD/T-ReX simulation for each protein target. The first energy function is identical to the force field (CHARMM22+CMAP with the GBMV2 solvent model) used to generate conformational decoys alternative to the starting ones. In addition, three statistical potentials were applied to rank order the generated conformers from the simulations. They included the RWplus energy function [34], the dDFIRE potential developed by Yang and Zhou [33], and the GOAP statistical potential developed by Zhou and Skolnick [35].

Generated conformers were analyzed in terms of their fraction of native contacts (*f*_N_). For a given decoy structure, the native contacts are identified as all side-chain center-of-mass pairs (*i*,*j*), such that *j*>*i* and whose distances are less than a cutoff of 6.5 Å. Using this approach for each decoy conformation, *f*_N_ is the number of native contacts in the decoy divided by the total number in the X-ray crystal structure.

## Results and Discussion

**Figure 1:**
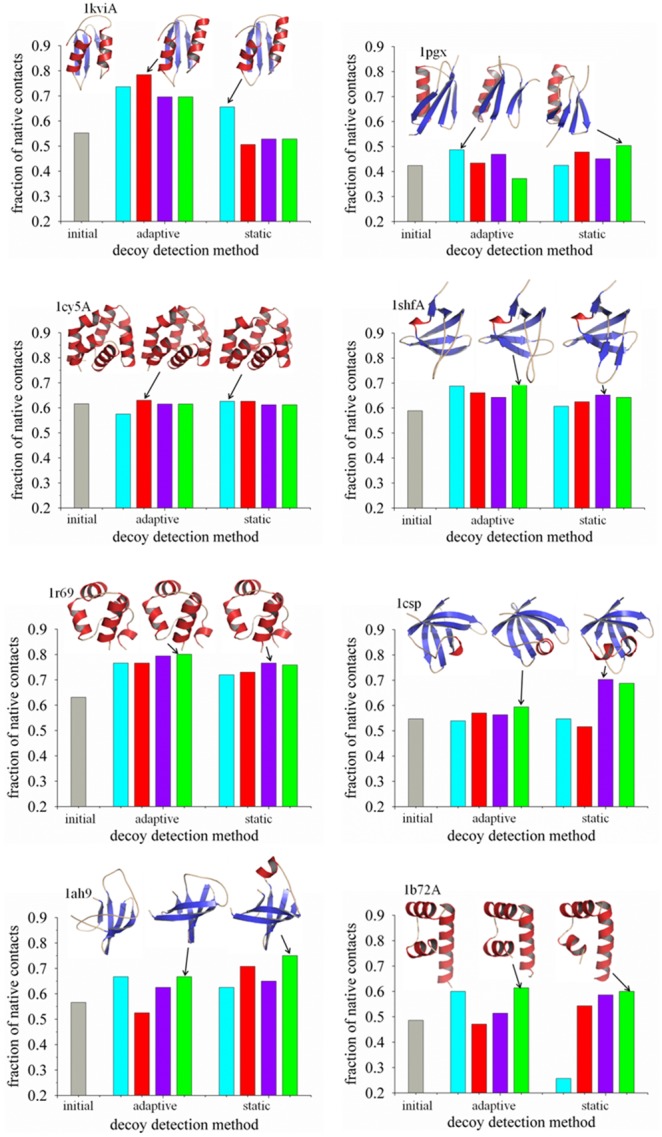
Comparison of adaptive and static T-ReX simulations for the refinement of protein target decoys 1kviA, 1pgx, 1cy5A, 1shfA, 1r69, 1csp, 1ah9 and 1b72A. A bar graph is shown of the computed fraction of native contacts for each detection method. Conformers were culled from the ensemble of replica clients at a temperature of 275-order rank detection is based on the energy functions CHARMM22/GBMV2 (cyan-colored bar), GOAP (red bar), dDFIRE (purple bar), and RWplus (green bar). For each target, the initial unrefined decoy is the conformer with the highest fraction of native contacts (grey bar). With the exception of GOAP scoring, refinement results using the static method for 1kivA, 1pgx, 1cy5A, 1shfA, 1r69 and 1b72A were taken from an earlier reported study.^5^ Molecular models are illustrated for the crystallographic structure and the top-ranked conformers from the adaptive and static sampling methods.

Table 1 presents the comparison between the adaptive and static T-ReX simulations for refinement of eight protein targets. Reported are the computed *f*_N_ values for the top-scoring conformations as detected by the four energy functions of CHARMM22/GBMV2, GOAP, dDFIRE and RWplus. Also included are *f*_N_ values averaged over the top-scoring 16 conformations for each scoring function. While there are various measures of rank order and similarity between conformers of a generated ensemble (e.g, global distance test scores, etc.), we selected to apply a straightforward approach based solely on the scoring functions and their relevance to detect high *f*_N_. For comparison purposes, the initial decoy set listed for each target is the *f*_N_ range for the starting 16 decoys. The simulation results are also illustrated in Figure 1, with *f*_N_ values that correspond to molecular model representations for each target, showing the X-ray crystallographic structure and the top-scoring conformers from refinement of the original decoys. The structural models demonstrate the diversity in the fold topologies among the set of targets and their conformational scoring.

**Table 1:** Fraction of native contacts computed from adaptive and static T-ReX simuations.*

Target	Initial range decoys	Adaptive Client Tempering				Static Client Tempering			
		FF	GOAP	dDFIRE	RWplus	FF	GOAP	dDFIRE	RWplus
1kviA	0.44–0.55	0.74 [0.68]	0.78 [0.68]	0.70 [0.70]	0.70 [0.70]	0.67 [0.62]	0.51 [0.58]	0.53 [0.59]	0.53 [0.61]
1pgx	0.31–0.42	0.49 [0.41]	0.43 [0.44]	0.47 [0.43]	0.37 [0.37]	0.43 [0.44]	0.48 [0.46]	0.45 [0.47]	0.50 [0.47]
1cy5A	0.50–0.62	0.58 [0.58]	0.63 [0.61]	0.62 [0.63]	0.62 [0.63]	0.63 [0.61]	0.63 [0.62]	0.61 [0.62]	0.61 [0.62]
1shfA	0.32–0.59	0.69 [0.67]	0.66 [0.67]	0.64 [0.68]	0.71 [0.67]	0.61 [0.57]	0.63 [0.63]	0.65 [0.61]	0.64 [0.63]
1r69	0.52–0.63	0.77 [0.75]	0.77 [0.77]	0.79 [0.78]	0.80 [0.77]	0.72 [0.64]	0.73 [0.73]	0.77 [0.77]	0.76 [0.77]
1csp	0.43–0.55	0.54 [0.61]	0.57 [0.57]	0.56 [0.60]	0.59 [0.57]	0.55 [0.59]	0.52 [0.58]	0.70 [0.70]	0.69 [0.69]
1ah9	0.37–0.57	0.67 [0.63]	0.53 [0.51]	0.63 [0.64]	0.68 [0.64]	0.63 [0.61]	0.71 [0.74]	0.65 [0.66]	0.75 [0.73]
1b72A	0.29–0.49	0.60 [0.47]	0.47 [0.47]	0.51 [0.58]	0.61 [0.58]	0.26 [0.50]	0.54 [0.56]	0.59 [0.58]	0.60 [0.60]

*Notation FF denotes the CHARMM22/GBMV2 force field. Tabulated for each target is the top-scoring conformer detected from the scoring functions plus an average of the top-scoring 16 computed structures (reported in brackets).

Of the multiple metrics to assess the accuracy of the simulations, the fraction of native contacts is perhaps one of the more stringent structural measures of native-like conformations. Quality assessment of the modeled structures can be further evaluated by the customary use of C_α_ root-mean-square deviation (RMSD); however, conformers rank ordered by this measure may contain poor side-chain packing. From the overall results, the adaptive T-ReX simulations for the aggregate dataset gives an average *f*_N_ value of 0.62, computed as a statistical average over the four energy methods using the top-scoring conformer detected by each energy function. As a comparison, the T-ReX simulations using a static temperature condition yielded a nearly equivalent average of *f*_N_ 0.60. The starting decoys for the targets prior to refinement have an average *f*_N_ of 0.51 for the top-ranked conformers using the RWplus energy function to rank-order structures. The overall range for the starting decoy set is a top value of *f*_N_ 0.64 (target 1shfA) to a low value of 0.29 (1b72A).

Among the energy functions, no single function consistently outperformed the others for all targets in detecting conformers with the highest *f*_N_. Nevertheless, for both the adaptive and static T-ReX simulations, RWplus yielded the highest average *f*_N_ of roughly 0.63, while the lowest is given by CHARMM22/GBMV2 with a value of 0.60. The average maximum *f*_N_ sampled from the simulations of all targets is 0.74 for the adaptive T-ReX and 0.72 for the static method. While these sampling excursions seem to approach the downhill refinement regime on the force-field potential energy landscape for both methods [7], [22], the conformational populations of these basins and their detection from the energy functions were disappointedly poor.

Using the alternative RMSD metric to assess refinement, the overall trend from the energy functions is similar to that of *f*_N_. The RWplus yielded the lowest-average C_α_ RMSD decoy detection of 2.6 Å for the adaptive T-ReX sampling and 2.2 Å for the static method. The lowest-RMSD values sampled overall from the simulations were 1.7 Å using the adaptive method and 1.6 Å for the static method. The starting decoy set of 16 conformers per target exhibited a net average C_α_ RMSD of 3.5 Å, with values ranging from 1.4 Å (target 1kviA) to 10.7 Å (1shfA). Collectively, the static method achieved lower RMSD values for the combined energy functions and, as illustrated below, this is related to the dynamics of heating and cooling clients by the adaptive method driven by the topology of the CHARMM22/GBMV2 energy landscape.

**Figure 2:**
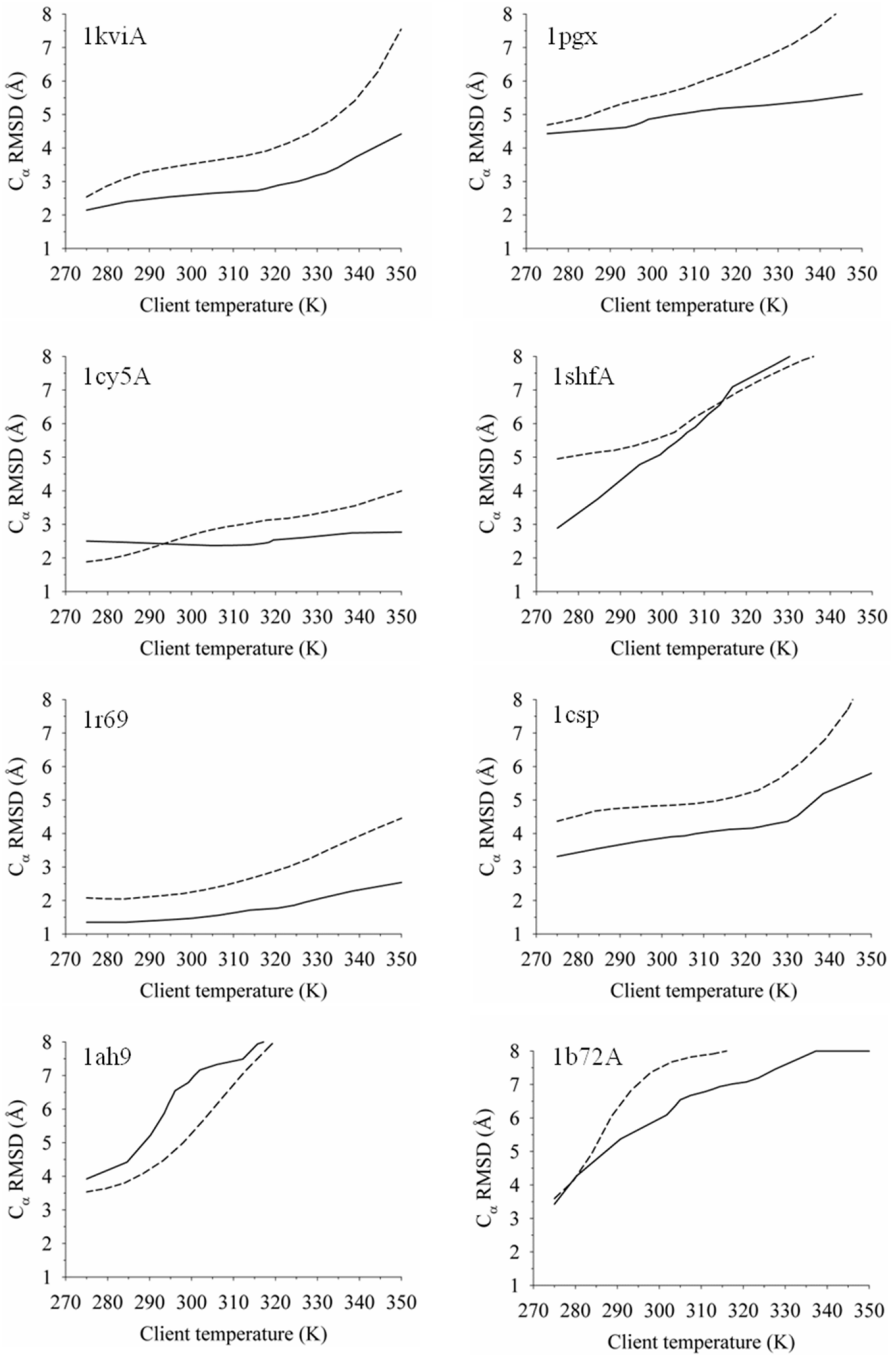
Determination by the adaptive and static T-ReX methods of an average C_α_-RMSD excursion computed as a function of client temperature for each protein target. The solid line denotes the adaptive sampling method and the dashed line represents the use of a static distribution of client temperatures.

To better understand the effects of temperature exchanges on conformational excursions, Fig. 2 shows a comparison between the adaptive and static T-ReX simulations in sampling C_α_-RMSD space as a function of client temperature. Since the adaptive method dynamically walks in temperature space, we selected to apply the final converged temperature set as an approximate of histograms over the evolved temperature path. The comparison of the two T-ReX methods shows in general the adaptive method produces a lower average C_α_-RMSD exploration and thus indicates less diverse sampling. Overall statistical fluctuations in the averages between the two methods are comparable (data not shown). Difference in excurions result follows from the diffusive flow of clients between the two temperature extremes and the enrichment of client populations near energetic barriers. Taken together, the adaptive method keeps structures near their starting decoys and leads to greater local optimization of conformational reorganization, rather than a more expanded search that is observed for the static method. Although there are undoubtedly pluses and negatives to a restricted local sampling, the escape from kinetic traps formed by misfolded side-chain packing may require more extensive excursions by way of unraveling the protein fold to achieve better results. While several targets show sharp funnels to lower RMSD values, many are gradual or, in the case of 1cy5A, nearly flat in the slope. The lack of steep funnels to low-RMSD values at *T*_min_ suggests difficulty in the force-field resolution to capture the downhill refinement regime.

One of the many factors that contribute to the resolution of the potential energy surface in sampling conformational space is the implicit solvent model. Depending on the protein target topology and the accuracy of the generalized Born model description, distortions in the population distributions have been noted, particularly for β-stranded proteins; see, e.g., [36], [37]. While generally an explicit solvent representation improves the sampling distributions for problematic cases using single-temperature simulations, challenges remain on implementation of a computationally efficient method for explicit solvent in replica-exchange simulations. To circumvent this problem, a hybrid explicit/implicit solvent model has been developed based on the idea of replacing the contribution of explicit solvent energies in the Metropolis exchanges with those of the GBMV2 model [7], [38]. This allows the number of replica clients in explicit solvent calculations to be the same as in the implicit solvent SGLD/T-ReX simulations while capturing the accuracy of conformational sampling on an explicit solvent landscape. Application of the hybrid solvent scheme with the static T-ReX method has been reported for the structure refinement of 1b72A and showed modest improvement over the implicit solvent model in obtaining a sharper funnel to low-energy native states [7]. Even with the improvement, detection remained roughly identical between the implicit and hybrid solvent simulation methods.

We next examine two targets in detail that illustrate the range of results from the adaptive T-ReX simulation method and its comparison with the static approach. Selection of these targets includes one where the adaptive method yields more accurate refinement and the other favors the static client approach. While these two test cases may appear anecdotal, they encompass a range of results that likely reflect a consensus from a much larger dataset in applying an adaptive scheme for temperature clients during a simulation trajectory.

**Figure 3:**
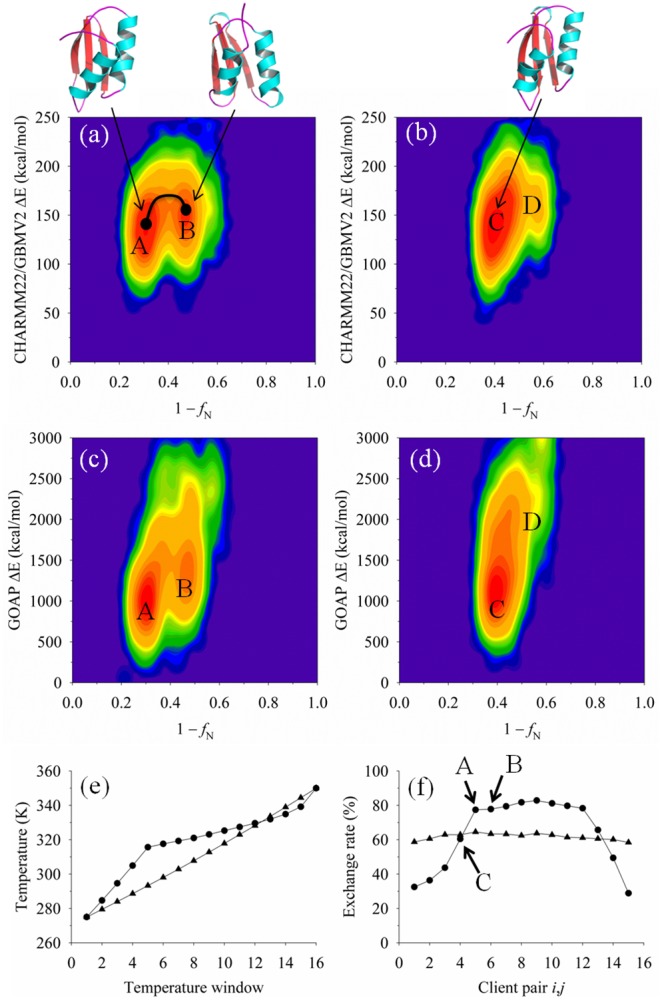
Conformational sampling of decoys for the target 1kviA, using the adaptive and static T-ReX simulation methods. Figures 3a–d show population-density profiles culled at 275 K from the trajectories generated by the two simulation methods. The energy functions CHARMM22/GBMV2 and GOAP are plotted as a relative change (ΔE) from a minimum value for each function versus the computed fraction of native contacts (*f*_N_). Population-density contours are displayed using a logarithmic distribution where the color range denotes the free-energy scale from high probability (red color) to low probability (blue color). Several highly populated energy basins are labeled in the profiles with representative conformers. Figure 3e shows the replica temperature windows (16 clients) as a function of the computed temperature at the end of 20 ns simulation time. Results from the adaptive method are shown as circle symbols and the static (geometrically fixed) temperatures as diamonds. Figure 3f reports the final replica exchange rate for each client pair *i*,*j* at 20 ns simulation time. Symbols of Fig. 3f are the same as given in Fig. 3e. Several basins highlighted in figures a-d are denoted by their client representation.

The first example given in Fig. 3 is the target 1kviA, which is a protein with a 2-layer αβ fold topology. Population-density profiles at 275 K are reported as a two-variable distribution of the conformational energy using the CHARMM22/GBMV2 function and the corresponding values of *f*_N_. In addition, the potential energies for both the adaptive and static methods are contrasted with GOAP scoring of conformers. It is observed that for this target, the adaptive T-ReX performed better at sampling the transitions between multiple energy basins (illustrated as basins A and B in Fig. 3a) than the static method of populating extensively one basin (labeled as C in Fig. 3b). Of the adaptive sampling method, CHARMM22/GBMV2 identified a conformer with a *f*_N_ of 0.74 (RMSD 1.8 Å) and GOAP a value of 0.78 (RMSD 1.7 Å), while for static method, the corresponding *f*_N_ values are 0.66 (RMSD 2.0 Å) and 0.51 (RMSD 2.0 Å), respectively. The top-ranked starting decoy had a value of *f*_N_ 0.60 (RMSD 2.2 Å).

Figures 3e and 3f illustrate the final temperature profiles of the adaptive and static T-ReX simulations after 20 ns for the target 1kviA. In comparison to the geometrically spaced fixed replica clients, the results of Fig. 3e show the dynamic walk in temperature space produces a sharp increase in the temperature at the lower end followed by smaller incremental changes in temperatures until reaching the upper end point. Whereas the geometrically spaced clients have exchanges in the range of 60% across the spectrum of nearest-neighbor clients and show no sharp unbalance, the adaptive method finds regions of energetic transitions to cluster clients to yield a significantly different profile. The range of temperatures from approximately 317 K to 330 K provides a thermal bath that allows the enrichment of nearest-neighbor client exchanges of roughly 80% (Fig. 3f), while both ends of the temperature condition drop off to roughly 30%. For this particular target, having a condition of high thermal convection among the clients leads to more efficient sampling of A
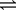
B transition separated at *f*_N_ ∼0.6 (Fig. 3a), whereas the static method becomes kinetically trapped. Figure 3f illustrates which clients are representative of basins A and B and their location on high-exchange manifold.

**Figure 4:**
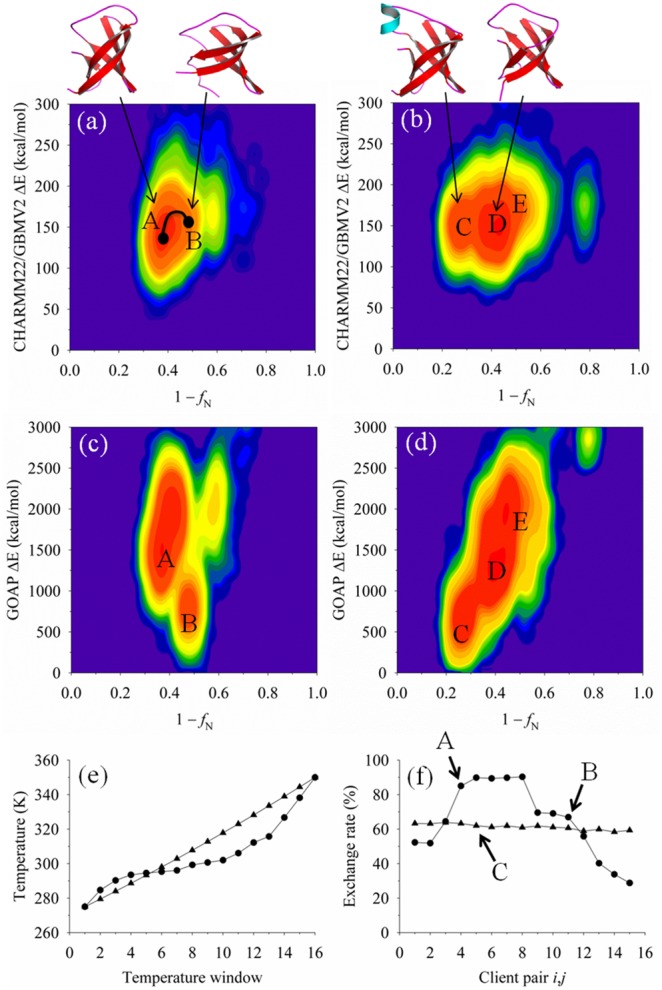
Conformational sampling of protein target 1ah9, comparing probability-density profiles computed by the adaptive and static T-ReX simulation methods. Detail descriptions of the plots are similar to those presented in Fig. 3.

The next target to illustrate is the protein 1ah9, an all-β protein of the OB-fold family. Unlike that of modeling 1kviA, the temperatures from adaptive sampling are generally lower than that applied through the static method (Fig. 4). This reduction in overall temperatures is comparable to that observed in the modeling study of SH3 [18] and is driven by the energetic and topological frustration of the CHARMM22/GBMV2 function in sampling structural reorganization of 1ah9. The cooler temperatures promote local optimization and falsely overpopulate the transition between A and B near-native basins (Fig. 4a), while in contrast the static method samples alternative states that are missing in the adaptive sampling (primarily basin C). Nevertheless, scoring structures by the CHARMM22/GBMV2 function yields similar conformers for the two sampling methods, namely the adaptive method identifies a *f*_N_ of 0.67 with a RMSD of 3.7 Å, and the static method yields *f*_N_ of 0.63 with a RMSD of 3.4 Å. As for overall excursions, the static method was able to sample a high *f*_N_ value of 0.82, while the adaptive approach was limited to *f*_N_ of 0.71. The lack of improvement in basin sampling from client exchanges evolving based on the energetic topology suggests that increasing temperature diffusion on a local transition identified to be overly favorable by the adaptive method can hinder the achievement of consistent results. In more broad terms, the bottleneck in the performance of adaptive temperature-based sampling is the limited large-scale structural reorganization, which is typically much slower than the rate of client exchanges that govern convection among the replicas [39].

The difference between the adaptive T-ReX and the static method for target 1ah9 is more pronounced when applying the GOAP energy function (Figs. 4c and d). The GOAP energy landscape for the static method shows a downhill refinement funnel that likely represents the all-atom resolution of the force field in sampling conformational space [7], [22], whereas the adaptive T-ReX reveals a landscape that favors a non-native collapsed basin. For the adaptive method, GOAP identified a conformer of *f*_N_ 0.52 (RMSD 5.6 Å), while the static T-ReX method detected *f*_N_ 0.71 (RMSD 1.7 Å). In comparison using the RWplus energy function, the adaptive and static methods differentiated *f*_N_ 0.67 (RMSD 3.7 Å) and *f*_N_ 0.75 (RMSD 1.6 Å), respectively. While the latter is significant refinement of the starting decoys that exhibited *f*_N_ of 0.57 as ranked by RWplus and consisted of RMSD values that ranged from 2.1 Å to 10.7 Å, there is a misfolded short helix in the topology identified by RWplus (Figs. 1 and 4).

**Figure 5:**
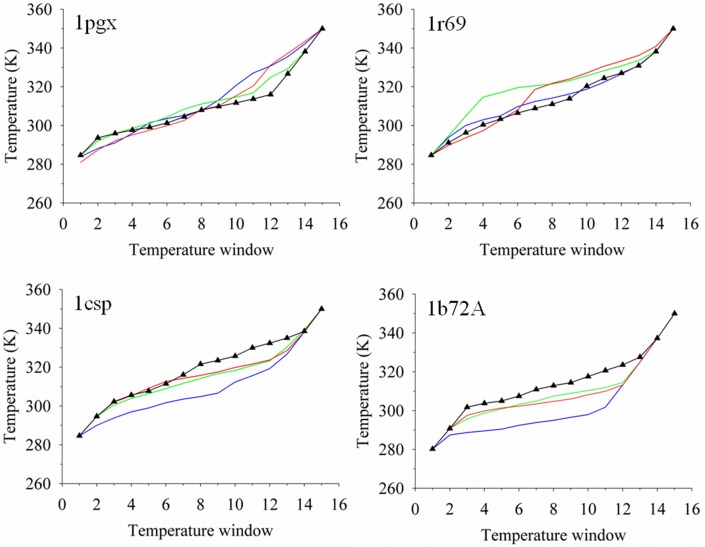
Dynamic temperature profiles for targets 1pgx, 1r69, 1csp and 1b72A. The color scheme represents simulation data taken from the trajectories at times of 5(blue line), 10 ns (green line), 15 ns (red line) and the final 20 ns (black line with symbols for each client).

One of the concerns about allowing the clients to dynamically walk in temperature space is the possibility of erratic tempering during the simulation trajectory. This is particularly important of a modeling approach that combines an adaptive scheme with the SGLD method of accelerating trajectories. Figure 5 shows snapshots of the temperature profiles in increments of 5 ns for targets 1pgx, 1r69, 1csp and 1b72A. We find that the clients evolve smoothly and there are no instabilities due to the SGLD method. Each profile is generally unique and the walkers in temperature space reflect the SGLD sampling of the protein conformational energy surface.

**Figure 6:**
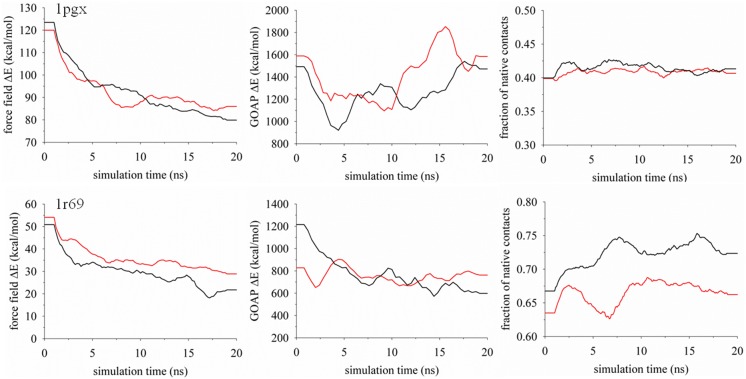
Energies and fraction of native contacts displayed as a function of the simulation time for targets 1pgx and 1r69. Shown are the computed energies from the CHARMM22/GBMV2 and GOAP potential energy functions, applied to scoring conformations extracted from the trajectory exchanged to the replica client at 275 K. The black colored line denotes results obtained from the adaptive replica-exchange method and the red line denotes simulations using the static temperature distribution. Data shown are running averages over 100–200 frames after the initial equilibration phase.

Related to issues of tempering stability is the concern of sampling exhaustiveness. Figure 6 shows the evaluation of three measures extracted from the replica client culled at 275 K along the simulation trajectory for targets 1pgx and 1r69. The measures are the CHARMM22/GBMV2 potential energy, the GOAP scoring of conformations and values of *f*_N_. As expected, values of CHARMM22/GBMV2 appear to gradually decrease due to continued optimization of bonded and non-bonded interactions during the simulations. In contrast, the character of the GAOP function varies widely among the simulation models and targets. In either model system, the simulations for 1pgx show slight optimization in *f*_N_ along the trajectory and suggests that refinement of the decoys is hindered by considerable frustration on the CHARMM22/GBMV2 potential energy surface. Conversely, the adaptive method for 1r69 finds the downhill refinement regime within roughly 7 ns and achieves rank-order uniformity in *f*_N_, even though the energies continue to evolve toward the optimization of structures in a native-like funnel. The final refinement for 1r69 clearly illustrates the potential advantage of the adaptive method.

## Conclusions

This work presented an assessment of an adaptive temperature-based replica exchange method for the structure refinement of a set of protein decoys. The difference between the conventional method of a static set of thermal clients and the adaptive method is that the latter allows clients to dynamically walk in temperature space near sharp energetic barriers that separate conformational basins. Unlike what is typically observed in protein folding-unfolding transitions, the energetic pathways between basins for structure refinement are less cooperative and show a reduced dimensionality in sampling the energy landscape given a starting decoy model with a near-native folded topology. It is of general interest to determine whether an adaptive T-ReX scheme provides any computational advantage in obtaining greater accuracy of refinement. Our study showed the adaptive method provided only minor improvement for refinement of protein models over the static approach in the sampling of basins composed of native-like side-chain contacts. The results also showed the need for improvement in the development of more accurate statistical potentials for detection of refinement extracted from all-atom simulations.
